# “Omics” and Immunologic Approaches to Optimizing Cure Rates in HER2-Positive Breast Carcinomas

**DOI:** 10.3389/fonc.2014.00334

**Published:** 2014-11-18

**Authors:** Elda Tagliabue, Manuela Campiglio

**Affiliations:** ^1^Molecular Targeting Unit, Department of Experimental Oncology and Molecular Medicine, Fondazione IRCCS Istituto Nazionale dei Tumori, Milan, Italy

**Keywords:** omics, HER2, targeted therapy, breast cancer, trastuzumab resistance

While clinical results indicate the benefit of HER2-targeted treatment, the fact remains that this therapy administered according to current FDA/EMEA-approved protocols can only cure about 50% of patients with HER2-positive breast carcinoma (1). Recently developed technologies in multi-omics analysis, together with a growing understanding of prognosis and predictive biomarkers, have provided significant advances in discovering and validating combination treatments that overcome trastuzumab resistance in HER2-positive breast cancer. However, several critical issues remain to be addressed. In this research topic, Herter-Sprie et al. (2) underscore the relevance of molecular alterations of HER2 that activate this molecule, including small insertions and missense mutations in the kinase domain, missense mutations in the extracellular domain, or large deletions of the extracellular domain in mechanisms of primary and acquired resistance to anti-HER2 therapeutics. In lung adenocarcinomas, 2–4% present activating mutations within the kinase domain of *ERBB2* analogous to those detected in EGFR and never coexisting with *EGFR, KRAS*, or *ALK* alterations (3–5). Such mutations mainly involve exon 20 and promote HER2 kinase activity. Another mechanism of HER2 activation consists in missense substitution clusters in subdomain II of the extracellular region with consequent mutation of residues important for stabilization of disulfide-bonded loops and formation of constitutively dimerized and activated receptor (6). Truncated HER2 proteins, which are predominantly found in breast cancers, derive from lack of substantial parts of the extracellular domain, either from proteolytic shedding of the ectodomain of the full-length receptor (p95HER2) or alternative mRNA translation from internal initiation codons (HER2 carboxyl terminal fragments) [reviewed in Ref. (7)]. In both cases, these forms generate activated homodimers maintained by intermolecular disulfide bonds and promote aggressiveness of breast cancer and resistance to trastuzumab because of the lack of a recognizable epitope.

The existence of activating mutations of ERBB2 and potential therapeutic options (e.g., afatinib or dacomitinib) to block their aggressive signal underscores the need for in-depth analysis of diagnostic properties specific for these alterations as part of clinical HER2 status evaluation before choosing among HER2-directed therapeutic options. In this context, Sapino and coworkers (8) present a thorough overview of diagnostic pathology instruments for the *HER2* gene and the protein, both at the technical and interpretational levels. In any given day of routine diagnostic practice, HER2 scoring depends on the demonstration of HER2 overexpression (by immunohistochemistry) or of *HER2* gene amplification by *in situ* techniques. Interpretation and reporting are crucial moments, especially in *in situ* hybridization, where amplification of the chromosome 17 centromeric region (CEP17) has been shownto account for misleading *HER2* FISH results, precluding anti-HER2-based therapy in some patients. In addition, heterogeneity of HER2 amplification must be considered in clinical therapeutic decisions, despite pilot results suggesting that a similar trastuzumab benefit for patients with HER2 amplification, either diffuse or focal (9). Another major debate surrounds the presence of full-length and truncated forms of the protein, with controversial clinical data reported on the therapeutic implications of these HER2 fragments and calling for the need to pathologically identify HER2 mutation carriers (10).

Molecular alterations of HER2 and its presence on the tumor cell membrane endow this oncoprotein with relevant immunological properties, making it an ideal target antigen for long-term cancer immunoprevention. As thoroughly described by Lollini et al. (11), HER2 is a class I oncoantigen with a causal role in tumor growth and progression and with a cellular localization accessible to recognition by T cells and to antibody binding. In studies to address cancer immunoprevention in HER2 transgenic mice harboring both activated (i.e., mutant) and wild-type rat and human oncogene, Lollini and coworkers (12, 13) developed antigen-specific cell-based vaccines able to elicit protective immune responses. Specifically, a triplex vaccine containing the p185 target antigen combined with two powerful non-antigen-specific stimuli, IL-12 and allogeneic class I MHC molecules, was highly protective against mammary tumor onset in HER2 transgenic mice. This vaccine also prevented metastatic outgrowth of tumors in mice bearing lung micrometastases indicating that the approaches developed for cancer immunoprevention could also be effective in cancer immunotherapy. Major long-term effects of vaccination were found to depend on the induction of T cell cytokines, in particular IFNγ, and of antibodies against the HER2 oncoprotein (14). A variety of immunologically based vaccines, such as those derived from cell proteins or peptides, DNA and virus, and dendritic cells, demonstrate the ability to prevent mammary carcinoma in HER2 transgenic mice, suggesting that their potential usefulness in adjuvant therapy of HER2-positive tumors to prevent the development of invasive breast cancer from HER2-positive DCIS as well as of metastases. As described by Marchini et al. (15), the efficacy of anti-HER2 DNA vaccination has been widely demonstrated in transgenic cancer-prone mice, which recapitulate several features of human breast cancers. Moreover, DNA vaccines are stable, relatively inexpensive, and simple to purify in large quantities, offering distinct advantages over other vaccine prototypes. Reportedly, the plasmid encoding both the extracellular and transmembrane domains of HER2 proved to be far superior to plasmids that only encoded the extracellular domain(secreted form) or the full-length protein in triggering protective immunity toward HER2-positive tumors (16 ). Moreover, the first 390 HER2 amino acids in rat are those responsible for inducing the protective immunity, mainly through anti-HER2 antibody production. Recently, the use of chimeric DNA vaccines derived by cloning the human cDNA fragment that encodes the first 390 NH_2_-terminal residues into the rat EC5-TM cut-down plasmid to regenerate the whole extracellular domain (HuRT) or by cloning the first 410 NH_2_-terminal rat residues into the remaining residues from human HER2 (RhuT) showed superior performance in breaking tolerance to the HER2 self-antigen (17, 18). Preclinical data obtained with these chimeric DNA vaccines have provided the rationale for their use in an ongoing Phase I clinical trial.

Overall, while the benefit of anti-HER2 therapies demonstrated in clinical trials indicates that HER2 is, to date, one of the most useful molecules for targeted therapy, optimization of these therapies for HER2-positive cancer patients awaits further studies. The issues considered in this Research Topic point to the need to better define the role of activating molecular alterations of HER2; refine HER2 clinical testing to design the most tailored treatment for breast cancer patients; and tailor anti-HER2 vaccines to prevent relapse in high-risk breast cancer patients or progression in patients with HER2-overexpressing minimal residual disease.

Such knowledge will also be highly relevant in considering the ability of new HER2-targeted drugs, i.e., the monoclonal antibody pertuzumab, the tyrosine kinase inhibitor lapatinib, and the antibody–drug conjugated trastuzumab-DM1, to improve the therapeutic effects of trastuzumab. While these new drugs also target HER2, it is not yet known whether activating HER2 mutations are sensitive to novel strategies. In view of recent results obtained in different clinical trials indicating that combining two anti-HER2 drugs with chemotherapy is the most effective treatment modality for HER2-positive patients (19), HER2 clinical testing to select patients for such treatments might be reconsidered. Indeed, the improved therapeutic benefit of combined anti-HER2 drugs might result in a lower threshold of oncoprotein expression levels than that required to obtain benefit from trastuzumab treatment. Finally, despite the improvements achievable with new anti-HER2 strategies, clinical practice shows that primary and/or acquired resistance to anti-HER2 strategies remains a relevant issue. Thus, development of effective vaccines for HER2-positive breast carcinoma to be used in addition to or integrated with already available drugs in patients with low-tumor burden and not heavily pretreated continues to be a distinct challenge.

## Conflict of Interest Statement

The authors declare that the research was conducted in the absence of any commercial or financial relationships that could be construed as a potential conflict of interest.
